# Adenosine diphosphate-ribosylation greatly affects proteins function: a focus on neurodegenerative diseases

**DOI:** 10.3389/fnagi.2025.1575204

**Published:** 2025-04-30

**Authors:** Chaowen Huang, Huilin Xiao, Yang Yang, Jiankun Luo, Yixi Lai, Shizhen Liu, Kanmin Mao, Jialong Chen, Liling Wang

**Affiliations:** ^1^Department of Respiratory Medicine, Jiangmen Central Hospital Affiliated Jiangmen Hospital of Sun Yat-sen University, Jiangmen, China; ^2^School of Public Health, Guangdong Medical University, Dongguan, China; ^3^Department of Rehabilitation, Affiliated Shenzhen Baoan Central Hospital Group of Guangdong Medical University, Shenzhen, China; ^4^Hubei Key Laboratory of Food Nutrition and Safety and the Ministry of Education (MOE) Key Laboratory of Environment and Health, Department of Nutrition and Food Hygiene, School of Public Health, Tongji Medical College, Huazhong University of Science and Technology, Wuhan, China

**Keywords:** adenosine diphosphate-ribosylation, neurodegenerative diseases, proteins function, PARP, post-translational modification

## Abstract

Adenosine diphosphate-ribosylation (ADPRylation) is a reversible posttranslational modification that plays a crucial role in cellular homeostasis and disease development. ADPRylation is produced via nicotinamide adenine dinucleotide hydrolysis and modifies proteins via corresponding transferases, mainly poly(ADP-ribose) polymerases (PARPs), the inhibitors of which have been used in the clinical treatment of cancer. ADPRylation is involved in various physiological processes, including pathogen infection, inflammation, DNA repair, and neurological disorders. In neurodegenerative diseases (NDs), dysregulated ADPRylation contributes to protein aggregation, neuroinflammation, and metabolic disturbances, while targeted modulation shows therapeutic potential. ADPRylation differentially regulates neurodegenerative processes, and PARP inhibitors can reduce neuroinflammation, oxidative stress, and metabolic dysfunction. However, challenges such as poor blood–brain barrier penetration and cell type-specific responses limit clinical translation. This review summarizes recent findings on the role of ADPRylation and PARPs in NDs, highlighting their involvement in protein aggregation and cellular signaling. It emphasizes the importance of ADPRylation in neuronal cells and supports the development of precision therapies targeting this pathway to address current treatment challenges in NDs.

## 1 Introduction

Cellular activity is fundamental to life and is primarily influenced by protein function and associated posttranslational modifications (PTMs) (Karve and Cheema, 2011). ADP-ribosylation (ADPRylation) is a reversible PTM that is widely involved in multiple critical biological processes, such as DNA damage, stress responses and cell proliferation, inflammation and cell death (Li et al., 2019; Kliza et al., 2021). Based on the number of ADP-ribose units attached to a target, the ADP-ribosylation modification is classified into mono-ADP-ribosylation (MARylation) and poly-ADP-ribosylation (PARylation), both of which are regulated primarily by poly(ADP-ribose) (PAR) polymerase family proteins (Groslambert et al., 2021). The human poly(ADP-ribose) polymerase (PARP) family consists of 17 members (Vyas et al., 2014; McGurk et al., 2019): (1) PARP-3, PARP-4, PARP-6, PARP-7, PARP-8, PARP-10, PARP-11, PARP-12, PARP-14, PARP-15 and PARP-16 are mono-ADPRylation transferases that transfer a single ADP-ribose from nicotinamide adenine dinucleotide (NAD^+^) to a target molecule; (2) PARP-1, PARP-2 and PARP-5 (tankyrase) add successive ADP-ribose units (2–200 units) to specific amino acids of proteins to form linear or branched polymer PARs (Aberle et al., 2020; Hendriks et al., 2021); and (3) PARP-9 and PARP-13, which have been reported to be enzymatically inactive, specific roles of which remain to be clarified. ADPRylation plays a crucial role in DNA damage repair and cancer therapy. Specifically, ADPRylation is involved in the regulation of DNA damage response pathways, where Mono-ADP-ribosylhydrolase 2 haploinsufficiency has been shown to impair the catalytic activity of PARP-1, resulting in chromosome instability and promoting tumorigenesis (Sakthianandeswaren et al., 2018). Additionally, nicotinamide adenine dinucleotide phosphate functions as an endogenous inhibitor of ADPRylation, suppressing PARP-1-mediated DNA repair and enhancing the sensitivity of tumor cells to PARP inhibitors (Bian et al., 2019). In the case of DNA damage repair, poly(ADP-ribose) polymerases (PARPs) function as DNA damage sensors, catalyzing ADPRylation at DNA lesions and functioning as docking platforms for DNA repair factors (Ray and Nussenzweig, 2017). PARP-1 has been reported to be the major PARP family member that is activated by single-strand DNA Breaks and double-strand DNA Breaks (DSBs) and interacts with X-ray repair cross-complementing protein 1 (XRCC1) to recruit the SSB repair machinery (Wu et al., 2021) or initiate the DNA-PKcs-KU70/80 signaling pathways to promote the repair of DSBs (Beck et al., 2014). PARs are removed from a protein by ADPRylation hydrolases. Each hydrolase carries specific domains and substrates. Poly(ADP-ribose) glycohydrolase (PARG) cleaves a PAR molecule that has bound proteins to free it or a single ADP-ribose moiety via its endoglycosidase or exoglycosidase activity (Houl et al., 2019). Furthermore, ADP-ribosylhydrolase 3 (ARH3) hydrolyzes the O-glycosidic bonds of short PAR chains or a single ADP-ribose moiety conjugated to a serine residue, in contrast to PARG, which less efficiently hydrolyzes a terminal ADP-ribose moiety (McGurk et al., 2019; Prokhorova et al., 2021). Humans express other ADPRylation hydrolases: Mono-ADP-ribosylhydrolase 1 and Mono-ADP-ribosylhydrolase 2 catalyze remove an ADP-ribose from acidic residues, and TADG1 ADP-ribosylhydrolase 1 (TARG1), Nudix Hydrolase 16 (NUDT16) and Ectonucleotide Pyrophosphatase/Phosphodiesterase 1 (ENPP1) reverse the MARylation or PARylation of proteins (Rosenthal et al., 2013; Sharifi et al., 2013; Palazzo et al., 2016; Zhang et al., 2020). Interestingly, hydrolases protect cells from excessive PARylation, which induces high levels of cytotoxicity. In fact, a PAR chain can be shuttled between the nucleus and cytoplasm, and cytoplasmic PAR is a fundamental factor in the abrogation of nuclear energy induced by BCL-2-Associated X Protein (BAX)-apoptosis inducing factor pathways, thereby linking PARylation to cellular apoptosis, also known as parthanatos (Fatokun et al., 2014; Mashimo et al., 2021). Hence, the dynamic and reversible progress of ADPRylation needs to be strictly controlled.

**Figure 1:**
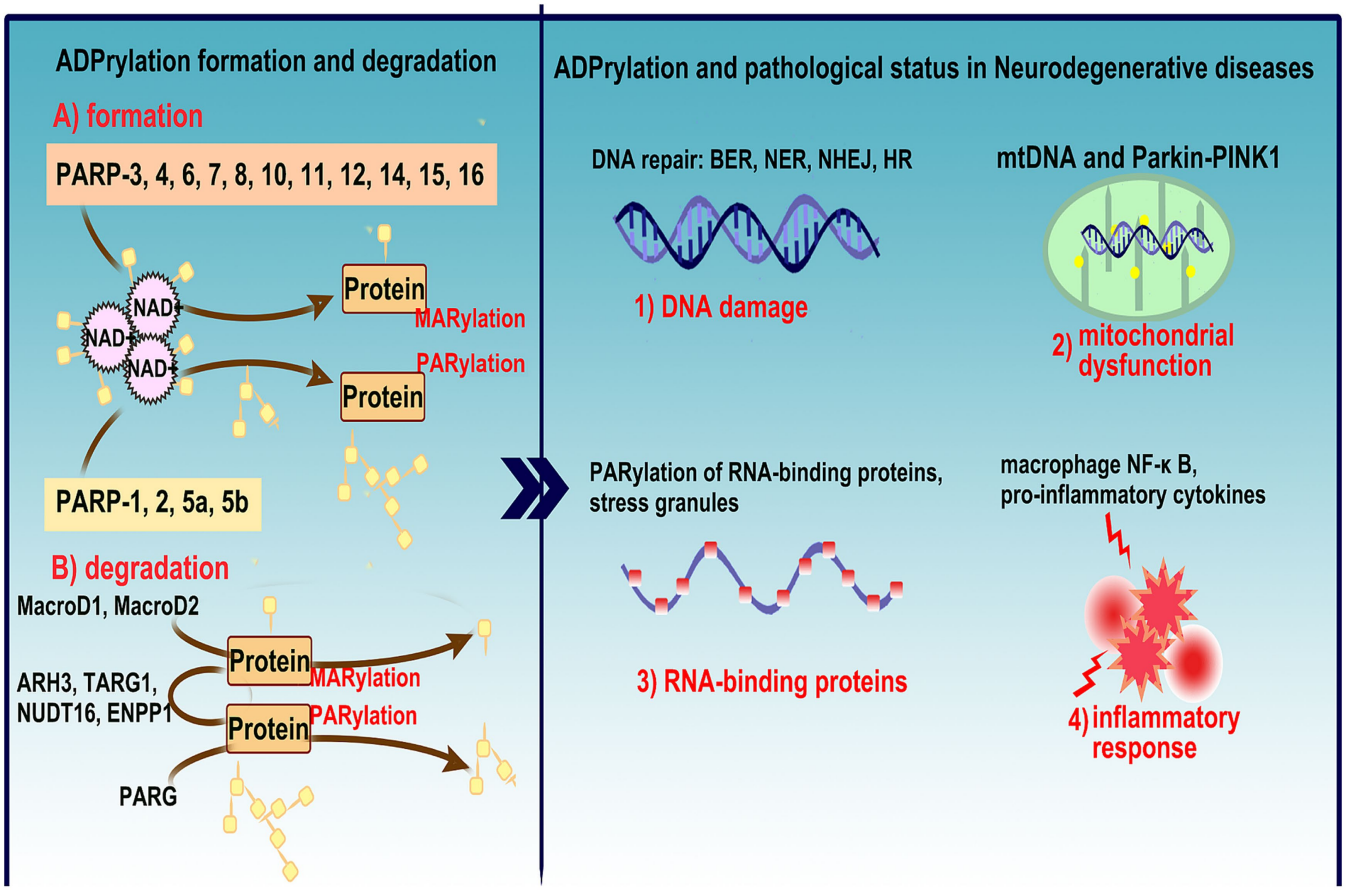
Overview of ADPRylation mechanisms and their role in NDs. This figure illustrates the formation and degradation of ADPRylation, a critical post-translational modification involved in various biological processes including DNA damage response, cell death, and inflammation. **(A)** Depicts the enzymatic activity of PARP family members (PARP-1, 2, 5a, 5b, and PARP-3, 4, 6, 7, 8, 10, 11, 12, 14, 15, 16), which transfer ADP-ribose units from NAD^+^ to target proteins via PARylation and MARylation, and the role of key hydrolases (MacroD1, MacroD2, ARH3, TARG1, NUDT16, ENPP1, PARG) in reversing these modifications, highlighting the dynamic and reversible nature of ADPRylation. **(B)** Links ADPRylation to the pathophysiology of neurodegenerative disease, discussing its impact on DNA repair mechanisms (BER, NER, NHEJ, HR), mitochondrial dysfunction via the mtDNA and Parkin-PINK1 pathways, and the implications for neuronal health. It also details the involvement of PARylated RNA-binding proteins in stress granule formation, activation of macrophage NF-κB pathways, and the release of pro-inflammatory cytokines, underscoring the comprehensive role of ADPRylation in exacerbating inflammatory responses within the nervous system. This figure encapsulates the complex interplay between ADPRylation and neurodegenerative disease progression, emphasizing its potential as a therapeutic target through the use of PARP inhibitors.

In recent years, the PARP-ADPRylation field has been further expanded by an increasing number of studies on aging-related diseases, especially neurodegenerative diseases (NDs), and it is now clear that PARylation of pathogenic proteins such as *α*-synuclein(α-syn) and *β*-amyloid (Aβ) can affect the phase separation and aggregation of these proteins, thereby accelerating disease progression (Strosznajder et al., 2012; Kam et al., 2018; Liu and Fang, 2019). In response to excessive PARP activity/ADPRylation that is evident in most brain regions and cerebrospinal fluid in patients with NDs, inhibition of PARPs attenuates mitochondrial damage and abnormal protein degradation systems (Lehmann et al., 2016; Mao et al., 2020), supporting the possibility that PARP/ADPRylation inhibitors protect neurons from pathological attack and contribute to normal brain function maintenance (Figure 1). Hence, this review is organized to present an overview of PARP/ADPRylation biochemistry and the known contribution of the ADPRylation modification to cellular signaling pathway activity in NDs, and then, future uses of PARP inhibitors as disease-targeting agents is discussed.

## 2 ADPRylation and DNA damage in neurodegenerative disease

Due to high oxygen demand and neuroelectrical activity, neurons are particularly vulnerable to reactive oxygen species (ROS) attack, leading to severe DNA damage (Belanger et al., 2011; Kharrati-Koopaee et al., 2021). Accumulating evidence suggests that DNA repair and genomic stability play crucial roles in aging-related NDs, including Alzheimer’s disease (AD) (Hou et al., 2018), Parkinson’s disease (PD) (Gonzalez-Hunt and Sanders, 2021) and amyotrophic lateral sclerosis (ALS) (Penndorf et al., 2018). Throughout their lives, organisms trigger a series of mechanisms to prevent genomes from being poisoned by endogenous and exogenous factors, and multiple monitoring and repair mechanisms have been acquired to repair DNA lesions in a timely manner. Many different NDs are characterized by significant overlap in signaling pathways and pathogenic patterns. Among neuronal DNA repair, base excision repair (BER), nucleotide excision repair (NER) and nonhomologous end joining (NHEJ) repair are considered major types, while homologous recombination (HR) is not typically induced (Lautrup et al., 2019; Mitra and Hegde, 2019; Gupta et al., 2021). However, recent studies have shown that HR, traditionally linked to the cell cycle, is also activated in post-mitotic neurons via RNA strand sensors, such as the RNA recognition motif of proteins like Fused in Sarcoma (FUS). This RNA-templated repair mechanism aids in fixing double-strand breaks using nascent RNA templates during transcription (Welty et al., 2018; Mamontova et al., 2023). PARP-1 plays a key role in regulating various DNA damage repair modalities, and its activation can hinder the pathological progression of NDs induced by environmental toxins. Methyl-4-phenyl-1,2,3,6-tetrahydropyridine (MPTP) is commonly used to establish animal models of PD (Przedborski et al., 2000; Cao et al., 2008). After MPTP exposure, mice show increased ROS and DNA damage together with hyperactivated PARP-1 in SNpc neurons (Wang et al., 2003). PARP-1 is involved in both classical NHEJ and alternative NHEJ repair mechanisms, as well as HR through the DNA-PKcs-KU70/80 signaling pathway (Wei and Yu, 2016; Noordermeer and van Attikum, 2019), whereas a portion of PAR molecules are labile in the cytoplasm, leading to apoptosis (Wang et al., 2016). The tumor suppressor p53 is heavily involved in the response to DNA damage. Upon activation, p53 accelerates cell cycle progression and induces apoptosis through the regulation of downstream genes like Bax, Fas, RB1, and Cdkn1a (Scoumanne et al., 2009; Jenkins et al., 2012; Iida et al., 2013). Interestingly, p53 itself is regulated by post-translational modifications, including phosphorylation and PARylation, which stabilize the protein and activate its tumor-suppressive functions (Fischbach et al., 2018). In the context of NDs, such as PD, p53 has been shown to be heavily PARylated by PARP-1 (Mandir et al., 2002), indicating a critical interplay between p53, DNA damage, and ADPRylation in neurons. In MPTP-induced PD models, the presence of p53 PARylation is consistent with the activation of cell death pathways and neuronal degeneration following DNA damage (Gonzalez-Hunt and Sanders, 2021). Moreover, familial PD is closely associated with mutations in the Synuclein Alpha (SNCA) gene encoding *α*-syn. SNCA A53T (Chen et al., 2020), A30P (Paiva et al., 2018), A30G (Liu H. et al., 2021), or E46K (Zhao et al., 2020) mutant mice present aggregated α-syn and Lewy bodies in dopaminergic (DA) neurons, disrupting the normal function of neurons and even leading to neuronal death. Recent evidence suggests that α-syn can directly interact with DNA, generating DNA strand breaks and activating PARP-1, further promoting neurodegeneration through the amplification of α-syn aggregation (Schaser et al., 2019). The subsequent DNA damage induces PARP-1 activation, and activated PARP-1 increases PAR recruitment to α-syn, thereby accelerating α-syn aggregation via the generation of additional toxic fibrotic structures. However, mono-ADP ribose does not interact with α-syn and exerts no toxic effect on α-syn (Kam et al., 2018). Collectively, these data indicate that the activation of PARP-1 in response to DNA damage and the subsequent PARylation of p53 appear to be central to the progression of several NDs, highlighting the importance of targeting PARP-1 and the ADPRylation pathway for therapeutic intervention. In addition, PARP-2, PARP-3 and PARP-10 have also been reported to be involved in DNA damage repair via ADPRylation (Beck et al., 2014; Nicolae et al., 2014), and whether they are involved in the development of NDs remains to be further investigated. Notably, mitochondrial DNA (mtDNA) can also be abrogated in response to damaged nuclear DNA, and this mitochondrial response is associated with NDs (Antonyova et al., 2020; Martin-Jimenez et al., 2020). Through nuclear-mitochondrial cross talk, PARP and ADPRylation are involved in mitochondrial NAD^+^ metabolism (Pang et al., 2018). PAR chain synthesis mediated through mitochondrial PARP-1 (Brunyanszki et al., 2014; Basello and Scovassi, 2015) supports the notion that the PARylation of mitochondrial proteins involved in mtDNA lesion repair contributes to organelle homeostasis.

**Figure 2:**
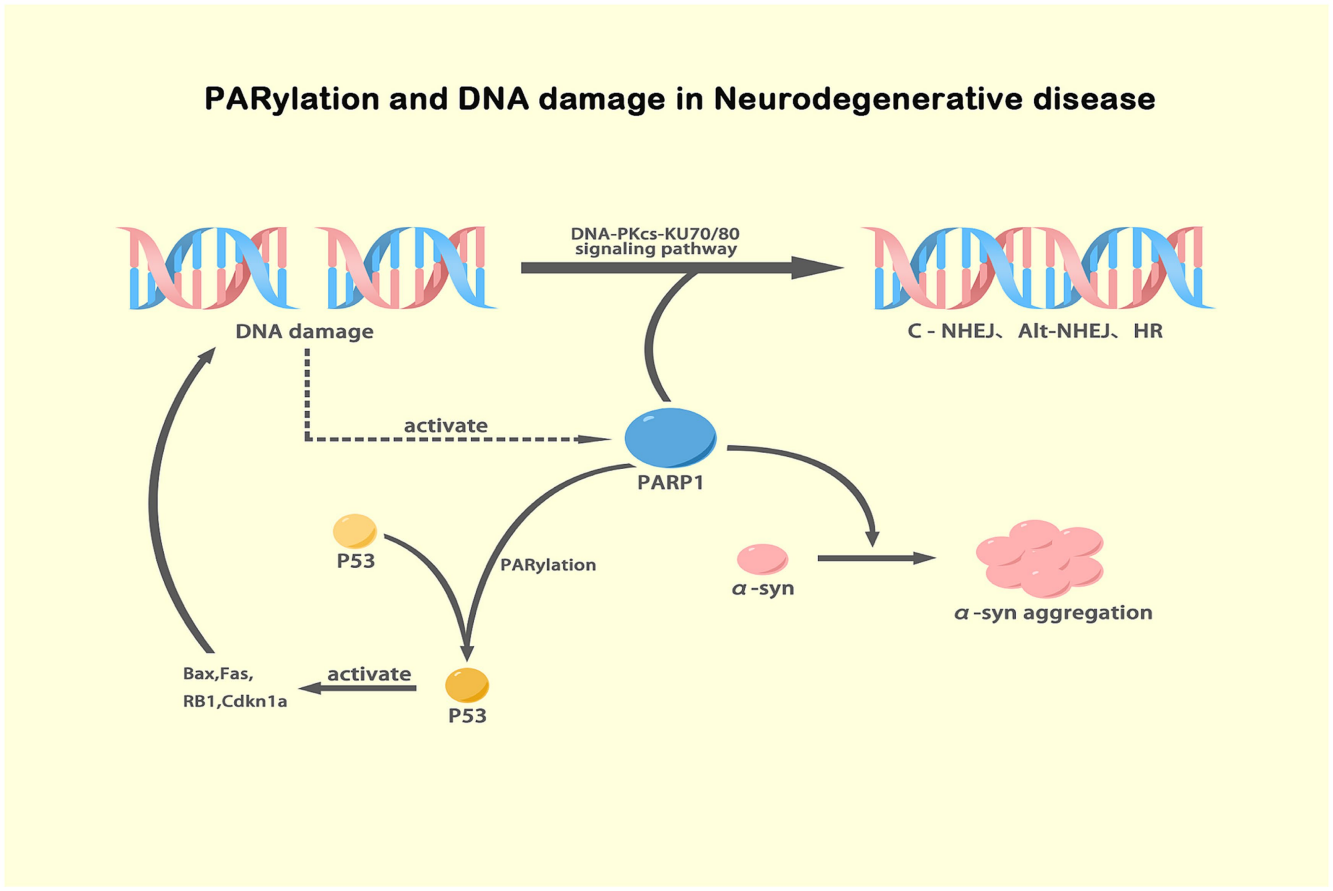
Mechanisms of PARP-1 activation and its effects in NDs. This illustration depicts the role of PARP-1 in DNA damage response where it activates DNA repair pathways including DNA-PKcs-KU70/80 signaling for classic and alternative NHEJ and HR. It also shows how PARP-1-mediated PARylation of p53 stabilizes and activates this protein, leading to the activation of apoptotic genes such as Bax, Fas, RB1, and Cdkn1a. Additionally, the diagram connects excessive PARylation of α-syn, driven by PARP-1 in response to DNA damage, to its aggregation, a key pathological event in PD. This comprehensive view underscores the dual impacts of PARylation in DNA repair and protein aggregation, highlighting the therapeutic potential of PARP inhibitors in NDs. Created with BioRender.

Huntington’s disease (HD) is an autosomal dominant disorder caused mainly by mutations in the Huntingtin (HTT) gene on Chromosome 4 (Bates, 2003). In neurons, the mutated HTT protein is widely expressed and aggregates, affecting the normal function of neurons and ultimately leading to death (Steffan et al., 2000). Relevant data show that HD patients carry mutations in Exon 1 of the HTT gene, thereby amplifying CAG repeats, which is associated with DNA repair mechanisms and enzymes that are similar to telomerase (Neueder et al., 2017; Gao et al., 2019). Mutations in DNA damage senor proteins, a class of proteins that monitor and detect DNA damage within cells, have been shown to reduce the stability of core checkpoint proteins (Mec1, Rad 9, and Rad 53), resulting in abnormalities in the recognition of CAG repeat sequences cells, leading to the increased frequency of CAG repeats (Massey and Jones, 2018; Maiuri et al., 2019). In addition, HTT can interact with a variety of transcription factors and thus affect protein expression (Thomas et al., 2013; Goodliffe et al., 2020). Cyclic AMP response element-binding protein (CREB) mediates the expression of cyclic Adenosine Monophosphate and calcium-dependent genes by binding to CREB-binding protein (CBP) (Screaton et al., 2004). However, aggregated HTT recruits CBP without CREB, leading to the deacetylation of the transcriptional complex and resulting in the failure of downstream gene expression (Chaturvedi et al., 2012; Jeong et al., 2012). Recently, PARP-1 inhibitors have been confirmed to modulate the localization and activation of phosphorylated CREB and CBP in distinct striatal subregions (Pramanik et al., 2014; Cardinale et al., 2015), which indicates their potential as effective treatments for HD (Figure 2).

## 3 ADPRylation and damaged mitochondria in neurodegenerative diseases

Mitochondrial dysfunction is the main cause of many neurodegenerative disorders. In both sporadic PD and familial PD, mitochondrial dysfunction is linked to damaged neurons and aggravated pathological progression (Moon and Paek, 2015). After MPTP or rotenone poisoning, mitochondria are profoundly affected by global oxidative stress and DNA damage (Sanders and Greenamyre, 2013; Cheng et al., 2021; Dionisio et al., 2021). Mitochondrial oxidative phosphorylation plays a crucial role in energy metabolism, and neurons are highly energy dependent (Sheng and Cai, 2012; Markham et al., 2014). The link between mitochondria and brain disorders has been extensively studied but it has not been clarified completely. In postmortem brain tissue from PD and AD patients, defective mitochondrial genome integrity, mtDNA depletion and mtDNA rearrangements have been identified (Pinto et al., 2013; Gurunathan et al., 2019). Moreover, an animal model of damaged mtDNA displayed DA neurodegeneration accompanied by motor disorder (Song et al., 2017), suggesting that NDs may be results of neuronal mitochondrial damage. Indeed, polymeric mtDNA mutation may result in the depletion of mitochondrial NAD^+^, thereby eliciting energy transfer chain breakdown (Lauritzen et al., 2021). As a major consumer of cellular NAD^+^ and a responder to DNA damage, mitochondrial PARP involvement in mtDNA repair and metabolism is undisputed. It has been shown that inhibiting PARP-1 reduces the integrity of the mtDNA genome and the expression of Cyclooxygenase 1, Cyclooxygenase 2 and NADH Dehydrogenase Subunit 2, subunits of the respiratory complex encoded by mitochondrial genes (Mohammed et al., 2020). PARylation helps regulate the expression of mtRNA repair proteins such as UNG1 and MYH1, as PARP-1 is located at the promoter of the genes that encode them (Lapucci et al., 2011; Mohammed et al., 2020). PARP-1 deletion may disrupt the epigenetic regulation of nuclear genes involved in mtDNA repair, thereby impairing mitochondrial homeostasis. In addition, it has been proposed that activated PARP-1 in patients with AD can block mitochondrial respiration and impair mitochondrial function by lowering the expression of peroxisome proliferator-activated receptor *γ* coactivator-1α (PGC-1α). NAD^+^ plays an important role in mitochondrial redox balance and the tricarboxylic acid cycle (Lu et al., 2019). Two consumers of NAD^+^ are PARP-1 and Sirtuin 1 (SIRT1), which function antagonistically (Krishnakumar and Kraus, 2010). PGC-1α is a core regulator of mitochondrial function and acts as a transcriptional coactivator with transcriptional activity and is subject to acetylation, which is tightly regulated by SIRT1 (Cui et al., 2021). Therefore, overactivated PARP-1 induces NAD^+^ deficiency, blocking SIRT1 deacetylase activity, which affects the acetylation status of PGC-1α and mitochondrial function. Familial AD is associated with aggregated toxic Aβ is also strongly associated with NAD^+^ consumption and PARP-1 activation (Martire et al., 2013; Yu et al., 2021). Studies by Yu YZ on Drosophila overexpressing Aβ showed that PARP gene mutation or pharmacological supplementation with NAD^+^ precursors rescued mitochondrial dysfunction, supporting the idea that PARP polymorphisms and NAD^+^ bioavailability are linked to Aβ toxicity and AD severity (Yu et al., 2021).

**Figure 3:**
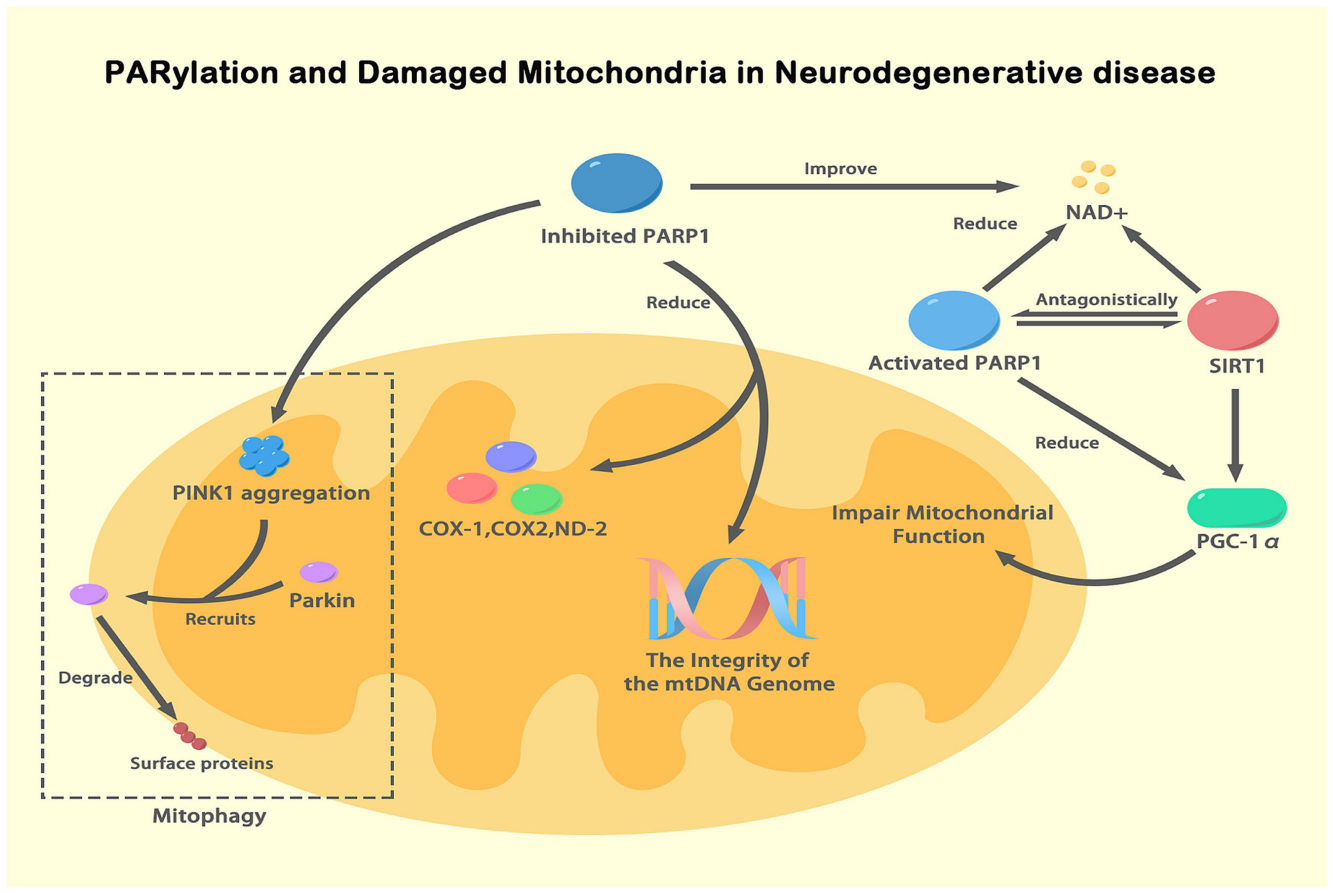
PARylation and damaged mitochondria in NDs. This figure illustrates the role of PARylation (a specific form of ADPRylation) in mitochondrial dysfunction associated with NDs. Activated PARP-1, in response to DNA damage, depletes NAD^+^ levels, impairing SIRT1 activity and the deacetylation of PGC-1α, thus reducing mitochondrial biogenesis and function. Inhibition of PARP-1 improves NAD^+^ availability, enhancing mitochondrial function and maintaining mtDNA integrity. NAD^+^ serves as a substrate for both PARP-1 and SIRT1, functioning antagonistically. PINK1 accumulates on the outer mitochondrial membrane under mitochondrial dysfunction, recruiting Parkin, which leads to the degradation of mitochondrial surface proteins and mitophagy. This figure highlights how PARylation, particularly through PARP-1, regulates mitochondrial function and integrity, contributing to neurodegenerative disease pathogenesis by influencing NAD^+^ levels and key mitochondrial processes.

Mitochondrial damage is closely related to altered mitochondrial morphology and abnormal mitochondrial dynamics, including processes such as mitochondrial fission, fusion, biogenesis and mitophagy (mitochondrial autophagy) (Lee et al., 2020). Among these processes, mitophagy clears and degrades damaged mitochondria and is a key mechanism in cellular homeostasis. Under pathological conditions, the mitochondrial membrane potential decreases, resulting in the inability of the mitochondrial kinase PTEN Induced Kinase 1 (PINK1) (Park6) to be properly sheared and degraded, and therefore, accumulates in the mitochondria. Aggregated PINK1 recruits the ubiquitin ligase E3 Ubiquitin Ligase (Parkin) (Park2) to the outer mitochondrial membrane, causing the degradation of mitochondrial surface proteins, which is required for the subsequent clearance of damaged mitochondria. This process is known as mitophagy (Jin and Youle, 2012; Lin et al., 2020). Dysregulated mitophagy, especially in long-lived cells such as neurons, promotes the development and progression of NDs. In addition, PINK1 and Parkin are known genetic factors in PD pathology. Mutations in PINK1 and Parkin result in impaired mitophagy and defective mitochondrial mass monitoring (Pickrell and Youle, 2015). Recently, inhibition of PARP has been shown to increase NAD^+^ levels and mitochondrial function in PINK1 or Parkin mutants (Yu et al., 2021). In Parkin-deficient models of mitochondrial dysfunction, decreasing PARP activity or increasing NAD^+^ bioavailability increased mitochondrial function and protected neurons from degeneration (Lehmann et al., 2016). Furthermore, in PINK1 mutants, increased oxidative stress and NAD^+^ depletion were associated with PARP activation. In contrast, mutation of PARP greatly attenuated the increase in protein PARylation and reversed the mitochondrial morphology defects and △ψm in the PINK1 mutant. PARP mutation is neuroprotective in the Drosophila PINK1 mutation model (Lehmann et al., 2017). Collectively, inhibiting PARP-1 may protect neurons from degeneration by regulating mtDNA and mitochondrial function (Figure 3).

## 4 ADPRylation and RNA-binding proteins in neurodegenerative diseases

ALS, also known as motor neuron disease (MND), is characterized by progressive muscle atrophy, feebleness and ultimately breathing failure (Al-Sarraj et al., 2011). To date, the most severely affected motor neurons in ALS are in the cortex, brainstem and spinal cord (Peters et al., 2015). Currently, the etiology of ALS is unknown, but 10–20% of cases may be associated with hereditary and genetic defects (Machado et al., 2019). More than 30 genes have been linked to ALS, with FUS, Superoxide Dismutase 1 and TAR DNA-binding Protein (TARDBP) genes being the three most common ALS-associated genes (Hou et al., 2016; Nakamura et al., 2020). When DNA damage occurs, FUS localizes to the site of the damaged DNA, is subsequently PARylated and then contributes to DNA damage repair, but FUS mislocalization expedites ALS progression in a PARP-1-dependent manner (Rulten et al., 2014). ADP-ribosylation has been confirmed to regulate several disease signaling pathways involved in the pathological progression of ALS, with the PARylation of RNA-binding proteins (RBPs) notable (Duan et al., 2019; McGurk et al., 2019). Some RBPs have one or more conserved RNA-binding domains (RBDs) and PAR-binding motifs (PBMs), explaining why PARP or PARylation impacts RNA homeostasis, such as through translocation and turnover (Forman et al., 2021). Notably, FUS is an RBP, and mutations in its PBMs may lead to aberrant phase separation and affect dynamic RNA interactions (Niaki et al., 2020). In addition, other pathological RBPs localized to ribonucleoprotein (RNP) granules in ALS are hnRNP A1 with a PARylation site at K298 and containing a PBM (Farina et al., 2021). Unexpectedly, changes in PARylation rates and PAR-binding levels of hnRNP A1 were found to be related, and hnRNP A1 mediated its own cytoplasmic translocation and association with stress granules based on these changes (Duan et al., 2019). Notably, PAR thus sequesters nontranslating mRNAs and increases aberrant protein aggregation by regulating disease-related RBPs.

**Figure 4:**
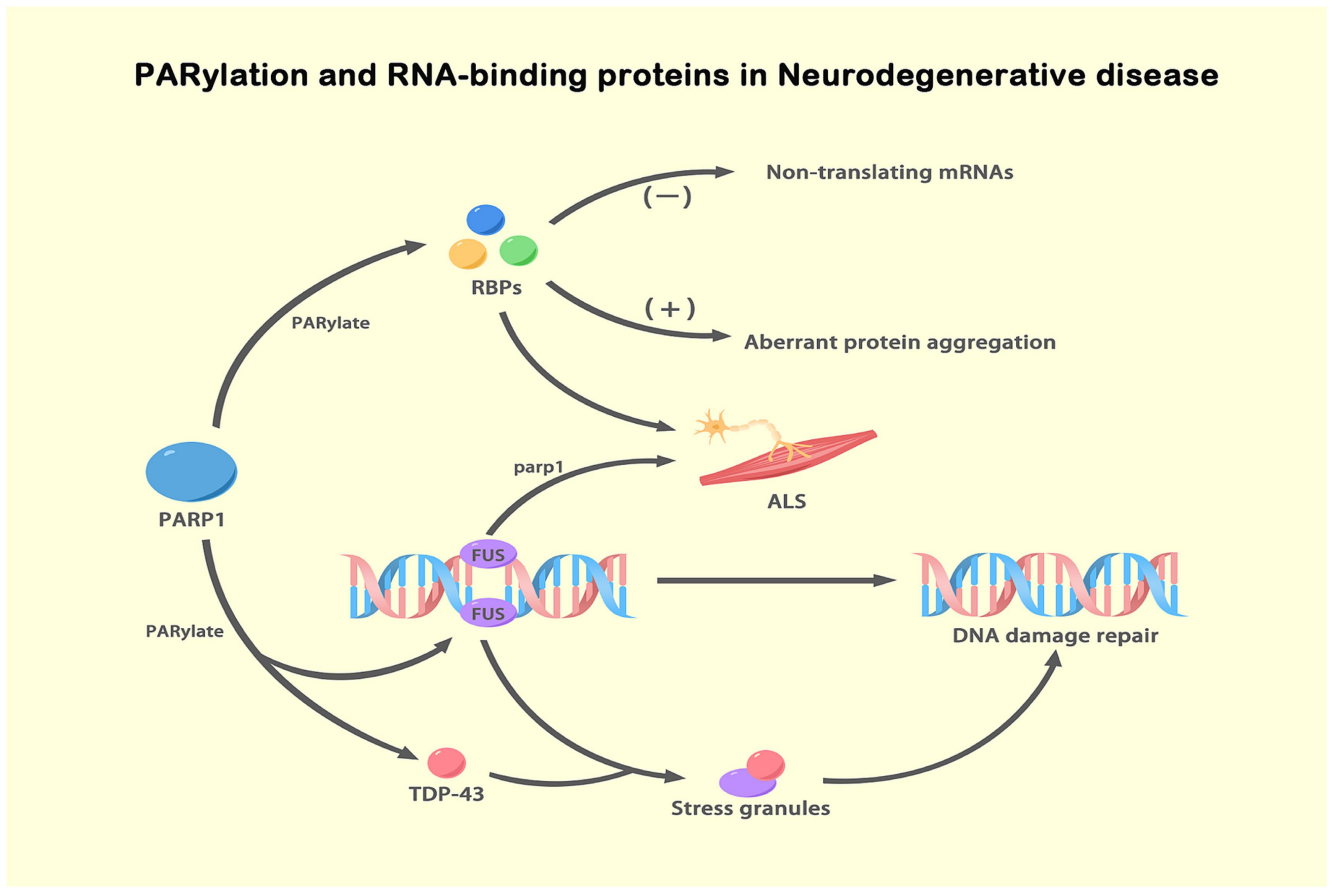
PARylation and RBPs in NDs. This figure illustrates the role of ADPRylation, specifically PARylation, in the regulation of RBPs and their impact on NDs, particularly ALS. PARylation, a specific form of ADP-ribosylation mediated by PARP1, modifies RBPs such as FUS and TDP-43. Upon DNA damage, FUS is PARylated by PARP-1 and recruited to DNA damage sites, facilitating DNA repair. Simultaneously, TDP-43 is PARylated and localizes to stress granules under stress conditions. This PARylation modification impacts RNA metabolism and protein aggregation, leading to aberrant protein interactions and the formation of toxic aggregates. These processes contribute to ALS pathogenesis. PARylation, through the regulation of RBPs, plays a critical role in maintaining genomic stability and regulating stress responses. The figure underscores the importance of PARylation in modulating key cellular processes involved in neurodegenerative disease progression.

Furthermore, the normal nuclear RNA-binding protein involved in transactive response (TAR) DNA-binding protein 43 (TDP-43, encoded by TARDBP) is a general pathogenic factor in ALS that is mislocalized to the cytoplasm, forming a ubiquitin-positive complex that is with hyperphosphorylated (Prasad et al., 2019; Chen, 2021). When DNA damage occurs, TDP-43 is recruited to the DNA lesion, facilitating the repair of the damaged DNA (Wood et al., 2020). Therefore, loss of nuclear TDP-43 and the subsequent increase in DNA damage is an early symptom of ALS. However, it remains to be determined how pathological TDP-43 is translocated to the cytoplasm. Leeanne McGurk reported that TDP-43 binds noncovalently to PAR at PAR-binding motifs, which are among the nuclear localization sequences om TDP-43; thus, PAR promotes TDP-43 accumulation by stimulating stress granule formation and liquid–liquid phase separation (McGurk et al., 2018a). In particular, under sustained stress, TDP-43 is phosphorylated and combines with other binding proteins, such as FUS, Atain 2 and hnRNPA1, to form stress granules that initiate pathological aggregation and promote progressive degeneration (Dao et al., 2019; Gasset-Rosa et al., 2019). Moreover, PARylation facilitates the progress of stress granule formation by mediating the condensation of the RNA–protein complex in a dynamic droplet, a process known as liquid–liquid separation (Leung, 2020; Jin et al., 2021). Finally, a small-molecule inhibitor of PARP-1/PARP-2 can block cytoplasmic TDP-43 foci and neurodegeneration in ALS (McGurk et al., 2018a). Similar to PARP-1/2, PARP-5a/5b activation-induced PARylation has also been revealed to aggravate TDP-43 expression in the cytoplasm and neurotoxicity (McGurk et al., 2018b). Collectively, the disease-linked pathologies related to FUS, hnRNP A1 and TDP-43 all highly rely on PARP-mediated nucleoplasmic transport and stress granules; therefore, agents that inhibit PARylation or PAR chain formation are therapeutic tools for ALS (Figure 4).

## 5 ADPRylation and inflammatory response

The PARP catalytic domain transfers the ADP ribose portion from NAD^+^ to the amino acid residues of a target protein, resulting in mono- or poly-ADP-ribosylation. Therefore, PARP members are “writers” of ADP ribose. Accumulating evidence shows that chronic inflammation is related to various pathological processes, including NDs (Gelders et al., 2018; Kwon and Koh, 2020) and aging (Bektas et al., 2018). A series of studies have shown that signal transduction pathways participate in the inflammatory response and are regulated by posttranslational modifications of proteins, such as ubiquitination, phosphorylation, methylation and acetylation (Chen et al., 2018; Francis et al., 2019; Leng et al., 2021). This section focuses on the relationship between ADPRylation and the inflammatory response and summarizes their roles in NDs.

**Figure 5:**
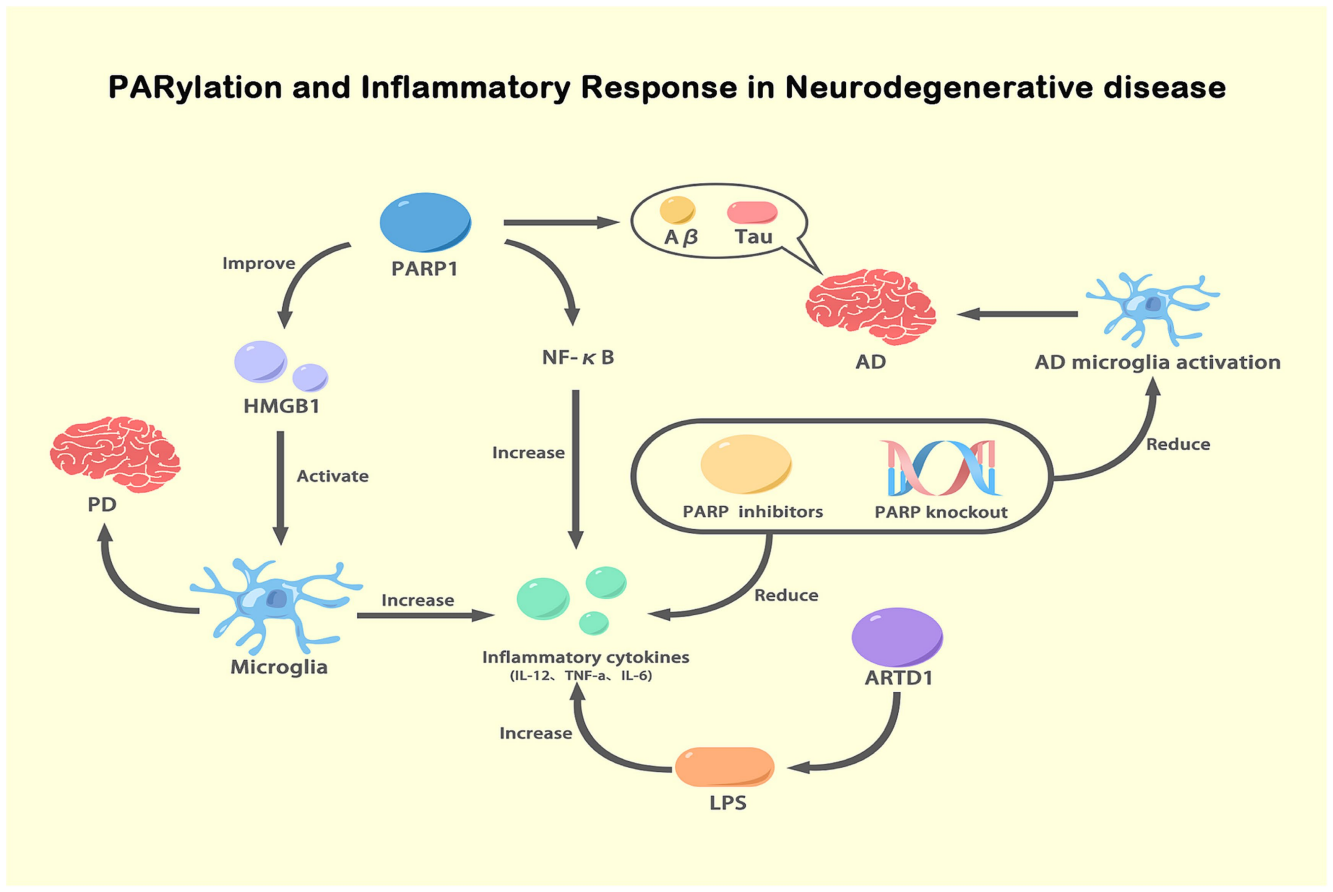
PARylation and inflammatory response in NDs. This figure illustrates the role of PARylation in modulating inflammatory responses in NDs. Activated PARP-1 enhances the expression and activity of NF-κB, leading to increased production of pro-inflammatory cytokines (IL-12, TNF-α, IL-6). These cytokines exacerbate inflammation and are involved in the pathogenesis of AD and PD. PARP-1 activation also promotes the release of HMGB1, which activates microglia, further increasing inflammation. In AD, PARP-1 activation is associated with the accumulation of Aβ and tau proteins, which activate microglia and contribute to disease progression. Inhibition or knockout of PARP reduces NF-κB activity and pro-inflammatory cytokine levels, thereby decreasing microglial activation and inflammation. Additionally, ARTD1, another ADP-ribosyltransferase, increases inflammatory cytokine expression in response to LPS stimulation. This figure highlights the critical role of PARylation in regulating inflammatory responses and its potential as a therapeutic target in NDs.

Some evidence suggests a potential connection between ADPRylation and inflammation. PARP-1 is associated with a proinflammatory response. In some cases, PARP-1 promotes an increase in the expression proinflammatory mediators, such as NF-κB, which do not depend on enzymatic activity, suggesting that PARP-1 is involved in the mechanisms underlying inflammation (Kauppinen and Swanson, 2007; Kim and Kang, 2019). Neuroinflammation plays an important role in the etiology of different nervous system diseases. Inflammatory responses in the nervous system are mediated by the inflammasome, activated immune molecules, and glial cells (Morimoto and Nakajima, 2019). However, chronic activation of inflammation can accelerates the progression of neurodegeneration (Beck et al., 2014). Alberto Chiarug reported that PARP-1 enzyme activity and PARylation are important to glial cell transcriptional activation and proved that PARP-1 activity-dependent regulation of NF-κB is a potential strategy for attenuating neuroinflammation and neurodegeneration (Komirishetty et al., 2017). In addition, PARP can regulate the levels of high mobility group box 1 (HMGB1). HMGB1 in the extracellular environment can activate microglia and increase the expression of inflammatory cytokines, which contribute to PD. Notably, anti-HMGB1 antibodies have been used to treat PD in mice (Yang et al., 2014; Sasaki et al., 2016). Moreover, the overactivation of PARP-1 in microglia directly impaired glutamate uptake, which is related to chronic neuroinflammation in NDs (Suh et al., 2007; Doaee et al., 2019). Various published reports have shown that PARP-1 inhibitors reduce the expression of proinflammatory cytokines, such as tumor necrosis factor *α* (TNF-α), interleukin-1 (IL-1) and IL-6, which manifests as a reduction in the mRNA levels of these inflammatory mediators (Liu Z. et al., 2021; Wasyluk and Zwolak, 2021). A recent study by Kunze et al. reported that ADP-ribosyl transferase diphosphate toxin like 1 (ARTD1) increased the lipopolysaccharide (LPS)-induced transcriptional upregulation of a series of genes, such as IL-12, TNF-a, and IL-6. These results emphasize the proinflammatory potential of PARP-1 (Love et al., 1999; Kunze et al., 2019). It has been shown that PARP interacts with Aβ and Tau in the brains of AD patients (Bagyinszky et al., 2020). Studies have confirmed that PARP-1 inhibition can slow the progression of AD (Salech et al., 2017; Hu et al., 2018). PARP knockout and treatment with PARP inhibitors can effectively reduce the inflammatory response caused by microglial activation and neurodegeneration in AD (Stoica et al., 2014; Mao and Zhang, 2021). Moreover, Abby L. Olsen proposed a PARP-1 inhibitor as a potential drug for PD treatment, but its mechanism of action is not clear (Olsen and Feany, 2019). The PARP-1 level is increased in PD models, including TgCRND8 mice with double Swedish (KM670/671/NL) and Indiana (V717F) mutations. Activated PARP-1 causes mitochondrial damage and directly modifies α-syn into more toxic strains, which accelerates the progression of PD (Kam et al., 2018). Recently, our team found that poly (ADP-ribose) polymerase 1 inhibition promoted α-syn degradation via transcription factor EB-dependent autophagy in a mutant α-synA53T model of Parkinson’s disease (Mao et al., 2020). Hitherto, extensive research on the relationship between ADPRylation and inflammation is still lacking, and we are actively working to make advancements in this field. As shown in our published data, we found that PARP led to the activation of NOD-Like Receptor Protein 3 by inhibiting autophagy, which exacerbated the progression of PD (Zhang et al., 2023; Figure 5).

## 6 Conclusion and future direction

In recent years, researchers have consistently suggested PARP activation plays an extremely important role in NDs, but its potential mechanism remains to be explored. In NDs, PARP-1 is activated due to the physiological characteristics of neurons and DNA damage, and α-syn undergoes pathological aggregation, disrupting the normal function of neurons and even leading to neuronal death. Mitochondrial oxidative phosphorylation plays a crucial role in energy metabolism, and neurons are highly energy dependent. Selective PARP-1 inhibitors, such as Olaparib, have been shown to reduce α-syn aggregation in PD models (Kam et al., 2018). However, it is important to note that PARP-5a/5b inhibitors may exacerbate the cytoplasmic localization of TDP-43 (McGurk et al., 2018b), highlighting that subtype selectivity is crucial in drug development. These findings suggest that PARylation or PARP-1 is involved in a widely altered DNA damage response in PD, indicating the therapeutic value of PARP-1 inhibitors. Mitochondria in brain tissue from PD and AD patients, defective mitochondrial genome integrity, mtDNA depletion and mtDNA rearrangements have been identified in patients with NDs. In addition, RBPs and the inflammatory response also play important roles in ADPRylation. It has been shown that inhibiting PARP-1 reduces the integrity of the mtDNA genome and the aggregation of pathological α-syn. In addition, it has been proposed that activation of PARP-1 is necessary for the formation of long-term memory, which is related to the integrity of nucleoli, rRNA and synaptic plasticity (Cohen-Armon et al., 2004; Goldberg et al., 2009). In the nuclei of hippocampus cells in AD patients, PARP-1 is lost, indicating that PARP-1 may be impaired in the function of the nucleoli. Some studies have suggested that PARP-1 causes cognitive impairment in AD patients for two reasons, one of which is because the loss of neuronal nucleoli PARP-1 may lead to synaptic plasticity defects, resulting in cognitive impairment; in the other possibility, in late stages of AD, PARP-1 may be overactivated and cause brain cell death (Zeng et al., 2016; Mao and Zhang, 2021). PARP-1 inhibitors can block the formation of long-term memory (Visochek et al., 2016), but treatment of AD patients with PARP-1 inhibitors has not been extensively studied. Recent advancements in PARP inhibitor development have demonstrated their growing potential for NDs treatment. Pharmaceutical companies and academic laboratories have identified novel brain-penetrant PARP inhibitors, further emphasizing the applicability of these compounds as potential therapeutics and imaging probes for NDs (Lengyel-Zhand et al., 2022). As a result, it is now timely to explore the possibility of repurposing these inhibitors for use beyond cancer treatment, particularly in the context of central nervous system (CNS) disorders where DNA damage and inflammation play significant roles. Specifically, parthanatos, a regulated form of necrosis associated with PARP-1 overactivation, is gaining attention as a critical target for intervention in CNS injuries (Mekhaeil et al., 2022). Parthanatos has been implicated in NDs, where the overactivation of PARP-1 leads to excessive ADPRylation, mitochondrial dysfunction, and neuronal cell death, particularly in response to oxidative stress and excitotoxicity (Zhang et al., 2025). As recent studies suggest, inhibiting PARP-1 in models of CNS injury, including traumatic brain injury and spinal cord injury, has demonstrated therapeutic promise by mitigating cellular damage and promoting tissue preservation (Berger et al., 2017). Therefore, integrating insights from parthanatos research into PARP inhibitor development could offer a new avenue for treating CNS disorders, positioning these inhibitors as dual-purpose agents for both therapeutic and diagnostic applications in NDs. However, the exact molecular mechanisms by which PARP-1 contributes to CNS development or to the progression of various NDs are incompletely understood. Due to its multifunctional nature, the biological pathways involving PARP-1 do not operate in isolation; instead, they run alongside other molecular processes, suggesting a complex interplay in regulating cell behavior and determining cellular outcomes. The comprehensive and in-depth study of the molecular mechanism underlying PARP-1 action is beneficial to the exploration of drugs in the treatment of NDs. PARP inhibitors have gradually shown potential for use in the treatment of NDs. The combination of further molecular mechanism exploration and clinical trials will provide strong evidence for their practical application in NDs treatments.
